# Data Sharing: How Much Doesn't Get Submitted to GenBank?

**DOI:** 10.1371/journal.pbio.0040228

**Published:** 2006-07-11

**Authors:** Mohamed A. F Noor, Katherine J Zimmerman, Katherine C Teeter

**Affiliations:** **1**DCMB Group/BiologyDuke UniversityDurham, North CarolinaUnited States of America

## Abstract

Funding agencies and journals require researchers to deposit DNA sequences in public databases such as GenBank when the paper is published, but how often do authors fail to do so?

A recent surge of interest in data sharing and data access has swept through the scientific community (e.g., [ 1]). Scientists recognize that free access to data is synergistic for fostering major advances. Concerns about standards of sharing are particularly acute with respect to large-scale DNA sequence and microarray data. Although some types of data have shallow histories or unclear protocols for how one would share them, DNA sequences have been deposited to the joint databases of GenBank, EMBL, and the DNA Databank of Japan [ 2, 3, 4] for over a decade, and many journals have policies requiring such submission before a paper can be accepted. For simplicity, we refer to these databases jointly as “GenBank.”

However, policies are only as good as their adherence and enforcement, and one should consider the effectiveness of past policies before launching new ones. Given its long history, an ideal case study is the deposit of sequences reported in published work into GenBank. We know from personal experience that authors of published papers reporting DNA sequences sometimes intentionally fail to deposit their sequences to GenBank and refuse to release them upon request. Is this a rare exception, or do many papers make it past coauthors, associate editors, editors, reviewers, and journal staff without providing the purportedly required data accession numbers?

We examined the frequency with which published studies failed to submit their DNA sequences to GenBank. We searched six journals that have explicit policies requiring the submission of DNA sequences ( Table 1). The previous six months (through February, 2006) of issues or most recent 30 papers presenting DNA sequence data were sought, though we examined a year of issues of one journal to test a longer-term trend (see below). Studies presenting only confirmatory sequence or sequences that would not be found in nature (e.g., transgenes) were not counted. We searched for GenBank entries using locus and species names, authors of the article, etc., thus executing a search that would mimic any scientist's approach.

**Table 1:**
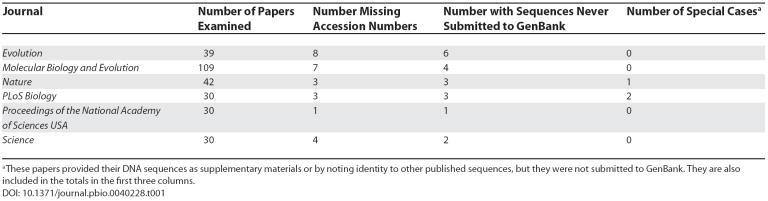
Submission of DNA Sequences from Published Studies to GenBank

No journal had complete compliance with its requirement for all DNA sequences to have been submitted to GenBank ( Table 1). Between 3% and 20% of papers in these journals did not include GenBank accession numbers, and between 3% and 15% of studies never submitted their DNA sequences at all. We also identified several papers with errors in the supplied accession numbers, but these errors were not counted. Table 1 also notes “special cases” that we considered less egregious violations of the journal rules. In these cases, the DNA sequences were noted in the paper itself, either as supplementary materials or by noting identity to published sequences.

The observation that some papers have sequences submitted to GenBank despite not providing accession numbers suggests that common reasons may be forgetfulness of the authors coupled with the lack of consistent policing at the journals. An author forgets to deposit the sequences, but then remembers (or is reminded) after the paper is published and submits them at that time. If this “oops effect” is common, we expect that the publication dates of the papers completely lacking GenBank submissions should be later on average than the publication dates of papers only missing accession numbers. We test this prediction with our observations from the journal *Molecular Biology and Evolution*, for which our survey spans a longer time period. Consistent with the prediction, we find a marginally significant difference in publication month ( *t* = 2.2, *p* = 0.08) in the expected direction between papers for which sequences were never submitted to GenBank and those for which sequences were submitted but accession numbers were not printed.

Our study concludes that the majority of papers provide their DNA sequences to GenBank. However, from the perspective of the journal, it is understandable how some papers get into print without providing sequence accession numbers. Paper submissions are closely checked for content by reviewers and associate editors, and they do not see it as their duty to police these policies—they merely evaluate the science and presentation. In contrast, the staff at many journals are often not scientists and not trained to recognize when and how these should be presented. Hence, although the staff may be asked to police this policy, it is understandable that they may miss several cases.

Although the failure to submit DNA sequences to GenBank appears rare, we must consider the consequences to an author if he/she intentionally publishes a paper without providing access to the data. There are two possibilities of how enforcement by the journal could be achieved. The author could be “flagged” such that future submissions to the journal would be declined until the DNA sequences have been released. This is a rather aggressive stance, and journals are unlikely to adopt it. We suggest an easy alternative. In the 21st century, many writers access publications online. We propose that, in cases where an author has not released DNA sequences, the author be given one month notice, at which point, if accession numbers are not provided, the publication is removed from the journal Web site until compliance is reached. One cannot take back the printed issue, but having the publication removed from the journal website would prevent anyone from accessing it online. This approach would focus the consequences to the deviant publication.

The databases of GenBank, EMBL, and the DNA Databank of Japan [ 2, 3, 4] serve as a model for data sharing from which the entire scientific community can learn. Although they sometimes get bad publicity for errors in DNA sequence submissions (e.g., see [ 5]), the positive impact they have had on all areas of biology is enormous. Let us look to the future and hope that new proposed forms of data sharing (e.g., [ 1]) are even more successful.
